# Bioavailable Microbial Metabolites of Flavanols Demonstrate Highly Individualized Bioactivity on In Vitro β-Cell Functions Critical for Metabolic Health

**DOI:** 10.3390/metabo13070801

**Published:** 2023-06-28

**Authors:** Emily S. Krueger, Laura E. Griffin, Joseph L. Beales, Trevor S. Lloyd, Nathan J. Brown, Weston S. Elison, Colin D. Kay, Andrew P. Neilson, Jeffery S. Tessem

**Affiliations:** 1 Department of Nutrition, Dietetics, and Food Science, Brigham Young University, Provo, UT 84602, USA; emilys.krueger@gmail.com (E.S.K.); jbeales@gmail.com (J.L.B.); tslloyd11@gmail.com (T.S.L.); nathansantiagobrown@gmail.com (N.J.B.); weston.elison@gmail.com (W.S.E.); 2 Plants for Human Health Institute, Department of Food, Bioprocessing and Nutrition Sciences, North Carolina State University, Kannapolis, NC 28081, USA; laura.griffin@taconic.com (L.E.G.); ckay@uams.edu (C.D.K.); aneilso@ncsu.edu (A.P.N.)

**Keywords:** gut microbiome, phytochemicals, flavanol metabolites, glucose sensitivity, responder analysis, personalized nutrition

## Abstract

Dietary flavanols are known for disease preventative properties but are often poorly absorbed. Gut microbiome flavanol metabolites are more bioavailable and may exert protective activities. Using metabolite mixtures extracted from the urine of rats supplemented with flavanols and treated with or without antibiotics, we investigated their effects on INS-1 832/13 β-cell glucose stimulated insulin secretion (GSIS) capacity. We measured insulin secretion under non-stimulatory (low) and stimulatory (high) glucose levels, insulin secretion fold induction, and total insulin content. We conducted treatment-level comparisons, individual-level dose responses, and a responder vs. non-responder predictive analysis of metabolite composition. While the first two analyses did not elucidate treatment effects, metabolites from 9 of the 28 animals demonstrated significant dose responses, regardless of treatment. Differentiation of responders vs. non-responder revealed that levels of native flavanols and valerolactones approached significance for predicting enhanced GSIS, regardless of treatment. Although treatment-level patterns were not discernable, we conclude that the high inter-individual variability shows that metabolite bioactivity on GSIS capacity is less related to flavanol supplementation or antibiotic treatment and may be more associated with the unique microbiome or metabolome of each animal. These findings suggest flavanol metabolite activities are individualized and point to the need for personalized nutrition practices.

## 1. Introduction

The human gut microbiome is comprised of ~100 trillion microbes and exhibits significant inter-individual variation in composition and function [1,2]. These microbes metabolize unabsorbed dietary components [3,4], forming metabolites which may manipulate host health [1,5,6]. Dietary flavanols, which are of interest for their chronic disease preventative properties, are also metabolized by the gut microbiome to more bioavailable forms [3,7]. Typically, these microbial metabolites are the major form of flavanols in circulation (based on circulating concentrations) and may be responsible for many of the effects of flavanol consumption observed in peripheral tissues [8]. The potential for the resultant flavanol microbial metabolites to promote host health outcomes is currently being explored, especially in the context of chronic diseases including diabetes, cancer, and obesity [9,10,11,12].

Dietary flavanols are a structurally diverse group of secondary plant metabolites found in foods such as fruits, vegetables, tea and cocoa (Supplemental Figure S1) [13,14,15,16]. Despite their abundance in the diet, the bioavailability of most flavanols is relatively poor [17]. Low-molecular weight monomers such as (+)-catechin and (−)-epicatechin are comparatively well absorbed in the small intestine [9,18]. High-molecular weight oligomers and polymers are poorly absorbed, however once metabolized by the gut microbiome their metabolites can be absorbed, deposited into circulation, and ultimately excreted in urine [19,20]. Flavanol feeding interventions in antibiotic (Abx) treated animals support this integral role of the gut microbiome in producing bioavailable and beneficial metabolites [1,21,22]. Evidence shows that bioavailable flavanol microbial metabolites may possess greater bioactivity than the native flavonoids [8,19,21,23]. Clinical research also connects various health outcomes to specific metabolite profiles, or metabotypes, stemming from the variability in microbiome populations and functions observed between individuals [2,24,25,26,27]. Health effects of the microbial metabolites are often stratified by metabotypes to elucidate relationships between microbial metabolites and the observed health outcomes [28,29,30,31]. In vivo and in vitro models of these metabotypes indicate that metabolites can beneficially modulate metabolism [7,8,19,20,21]. 

This growing body of evidence suggests that microbial metabolites may be the key by which flavanol intake modulates chronic diseases risk; however, current data present limitations [16]. Most in vitro microbial metabolite studies rely on commercially available purified metabolites which do not adequately recapitulate the complex physiological mixtures of compounds generated by the gut microbiome in vivo. We previously investigated the profiles of bioavailable native and metabolite compounds in rats treated with or without Abx and supplemented with high molecular weight flavanol rich grape seed extract (GSE), low molecular weight flavanol monomers catechin and epicatechin (C/EC), or a control [22]. Urinary flavanol metabolites (valerolactones, phenylalkyl acids, and hippuric acids) were associated with reduced cancer cell proliferation in vitro [22]. While We previously observed several metabolite bioavailability and bioactivity phenotypes between the experimental animal groups in the context of uroepithelial cell cancer, and therefore in the present study we investigated if the same metabolite mixtures impact the β-cell insulin secretion pathway in vitro [22].

Dysregulated insulin secretion by pancreatic islet β-cells is a key pathological characteristic of diabetes [16,32]. Under healthy conditions, β-cells secrete low levels of insulin during the unfed state when blood glucose levels are low (below ~5.6 mM) and considered non-stimulatory, while postprandially elevated blood glucose levels (~12 mM) stimulate insulin secretion in high levels [33,34]. Pathogenic β-cell function can result from a mismatch between blood glucose and insulin secretion, such as elevated insulin secretion during low glucose conditions or limited secretion during high glucose conditions. Glucose responsivity is measured in β-cells with the glucose stimulated insulin secretion (GSIS) assay [35,36,37]. The four common GSIS parameters include insulin secretion under low glucose exposure, insulin secretion under high glucose exposure, insulin secretion fold induction, and total insulin content of the β-cells. In the present study, we used GSIS to investigate the glucose responsivity of INS-1 832/13 β-cells cultured with the extracts of urinary flavanol metabolite mixtures collected previously [22]. While some studies demonstrate that flavanols may impact β-cell function, this cellular model is less commonly used than others such as adipose and skeletal muscle [8,33,34,38]. Building on promising in vitro, in vivo, and human studies [39,40,41,42,43], our objective was to investigate the bioactivity of the previously collected urine metabolite mixtures on β-cell GSIS capacity.

## 2. Methods

### 2.1. Animals and Diets

All animal work was approved by the Institutional Care and Use Committee of the David H. Murdock Research Institute (protocol #19-005) and was described previously [22]. Outbred male Wistar rats (*N* = 28) age 6-weeks were obtained from Charles River (Wilmington, MA, USA). Rats were individually housed under standard conditions (12-h light/dark cycle, 30–70% relative humidity, 20–26 °C) with *ad libitum* standard chow and water. After receiving the animals, the rats were acclimatized for 5 days on standard chow. Following acclimatization the rats were transitioned to the AIN-93G Growing Rodent Diet (D10012G, Research Diets Inc., New Brunswick, NJ, USA). Rats were randomly assigned to receive either grape seed extract (GSE), 1;1 catechin/epicatechin mixture (C/EC), or the vehicle control (water). The groups where further divided to receive either no Abx or an Abx treatment, forming a total of 6 experimental groups (*n* = 4–5 rats/group) [22].

### 2.2. Antibiotics (Abx) Treatment

A cocktail of 0.5 g/L vancomycin (Triangle Compounding Pharmacy, Cary, NC, USA), 1 g/L neomycin sulfate (Huvepharma, Maxton, NC, USA), 1 g/L metronidazole (Unichem Laboratories, Hasbrouck Heights, NJ, USA), 1 g/L ampicillin (Auromedics Pharma, East Windsor, NJ, USA) was administered in drinking water of all Abx treatment groups as described previously [21,22]. 

### 2.3. Flavanol Standards and Extracts

The preparation and oral gavage (Kent Scientific, Torrington, CT, USA) of the flavanol supplements Vitaflavan^®^ GSE (DRT Neutraceuticals, Dax, France), (+)-catechin hydrate (C) and (−)-epicatechin (EC, Millipore Sigma, Burlington, MA, USA) mix are described in the previous study [22]. The composition of the GSE is reported in Supplementary Table S1. Each supplement used gavage solutions composed of 125 mg/mL total concentrations and water was used as a vehicle control. GSE was administered to provided C/EC in monomer and mixed oligomer form. Rats were gavaged 1 mL of the assigned gavage solutions, independent of body mass, to provide the same total dose. Rats therefore received either GSE (125 mg total flavanols in the form of mixed flavanol monomers and oligomers), C/EC (125 mg total flavanols in the form of 62.6 mg C + 62.5 mg EC), or vehicle (0 mg flavanols).

### 2.4. Flavanol Metabolite Collection and Purification

After overnight fasting, treatments were administered by a single, acute oral gavage and urine was collected via a metabolic cage for 48 h as described previously [22]. Urine samples were frozen at −80 °C and rats were euthanized at the conclusion of the study. Water was removed by freeze-drying. Lyophilized urine solids were reconstituted with 5 mL of 0.1% formic acid in 1:1 methanol:ethanol, and then vortexed, sonicated, and centrifuged to precipitate out urea and salts. Supernatants were 0.22 micron sterile filtered (Sigma, Burlington, MA, USA), speedvapped to dryness (Thermo Fisher, Waltham, MA, USA), and stored at −80 °C. Prior to cell culture treatments, the samples were all reconstituted to the same volume (representing the approximate average urine volume) with sterile double-distilled water. All rats received the same dose and urine extracts were reconstituted to the same volume, representing a 1x concentration relative to the average urine volume. Thus, urine extracts normalized excreted compounds concentrations, eliminating urine volume as a variable, as our objective was to obtain the pool of total excreted (i.e., bioavailable) metabolites, independent of urine volume. 

### 2.5. Metabolite Profiling

Native flavanols and their microbial metabolites in the urine derived samples were quantified by LC-MS/MS as described previously [22]. Compounds determined *post hoc* as not applicable due to lack of presence in the samples, and those with poor linearity for external standards, were excluded from data analysis. Compounds presenting signals with intensities at or below the lower limits of detection were reported as 0 μM. Peaks with intensities between the lower limits of detection and quantification were reported as 0.0001 μM. After performing these adjustments, a total of 75 compounds were further analyzed. These compounds were classified based on the three categories: (1) native flavanol or flavanol microbial metabolites, (2) unconjugated form or Phase-II conjugated, and (3) metabolite stage: native flavanol, valerolactone, phenylalkyl acid or related, cinnamic acids or related, benzoic acids or related, hippuric acids or related, other aromatics, and non-aromatics.

### 2.6. Cell Culture

The rat derived INS-1 832/13 β-cells were maintained in RPMI 1640 + L-glutamine (Corning, Glendale, AZ, USA) with 10% fetal bovine serum (Sigma, Burlington, MA, USA), 50 µg/mL streptomycin and 50 U/mL penicillin (LONZA, Morristown, NJ, USA), 10 mM HEPES (Thermo Fisher, Waltham, MA, USA), and INS-1 832/13 supplement as previously described [8,44]. Cells under 100 passage doublings were plated in 24-well culture plates at ~1.05 × 10^5^ cells/mL (VWR, Radnor, PA, USA). Twenty-four hours after plating, cells were treated with the reconstituted urine derived extract samples diluted in media. Final treatment concentrations represented 10–100x dilutions relative to urine levels (due to the relative concentration from blood to urine) to approximate physiologically relevant circulating metabolites in vivo. Control β-cells were cultured with sterile double-distilled water equal to the value added for the 10% metabolite treated samples. After 24-h culture treatments, GSIS assays and harvesting were performed.

### 2.7. Glucose Stimulated Insulin Secretion (GSIS)

A static GSIS assay was performed on all treated cells as described previously [8,45,46,47,48,49,50]. Treatment media was washed from the cultured cells and replaced with secretion assay buffer containing low glucose (2.8 mM) to replicate the unfed state. Cells were acclimatized to the low glucose condition for 2 h followed by 1-h static stimulations in low glucose and then in high glucose (12 mM) to replicate the postprandial state. Insulin secretion samples were collected at the conclusion of the low and high stimulation periods. Finally, cells were lysed and harvested for total insulin content. Unstimulated, stimulated and total insulin were measured by ELISA [45,51] and normalized to total protein content measured by the bicinchoninic acid assay (Thermo Fisher, Waltham, MA, USA). Fold induction was calculated by dividing stimulated insulin secretion by unstimulated insulin secretion. Glucose responsiveness of cells was validated by measuring fold induction greater than 2 in vehicle treated β-cells (Supplemental Figure S2). To account for variation in β-cell glucose responsiveness at baseline, all results were normalized to the accompanying per plate vehicle controls, thus all insulin secretion and content results being are reported as relative values which are comparable across experiments. 

### 2.8. Statistical Analysis

Urine composition: To determine statistical significance of the main effects (flavanol and Abx treatment) and interactions, the concentrations of measured compounds in urine samples were compared by 2-way ANOVA. If a significant main effect or interaction was detected, Holm-Sidak *post hoc* tests to account for multiple comparisons were performed to determine differences among the 3 flavanol treatments within each antibiotic treatment group (Control and Abx). Holm-Sidak post hoc tests were also performed to determine differences between antibiotic treatments (Control and Abx) for each flavanol. The overall family-wise error rate was set as 0.05, with one family per group.

Treatment-level comparisons and individual-level dose responses: As indicated in the figure legends, means of treatment groups (*n* = 4 or 5 animal biological replicates represented as dots) were compared by 2-way ANOVA with Šidák’s *post hoc* test and presented in the treatment-level comparisons graphs. For the individual-level dose responses, the means of individual experiments (*n* = 3 β-cell culture biological triplicates represented as dots) were compared by 1-way ANOVA with Dunnett’s *post hoc* test. Error bars indicate standard deviation (SD). GraphPad Prism 9 (GraphPad, La Jolla, CA, USA) was used for all statistical analyses. Values of *p* < 0.05 was defined as significant *a priori*.

Responder vs. non-responder analysis: Analyses were performed to determine predictive relationships between urine composition and GSIS capacity. Due to the large number of doses and extensive list of compounds, individual compounds were not analyzed for correlations with activity and only data from the 5% urine dose (which showed the most significant effects) were employed for responder analyses. Associations between groups of compounds and inhibitory activities were performed for groups of compounds to establish general relationships between compound classes and activity. The data points were the calculated composition values for each urine sample, and the mean GSIS values for that urine sample. Error bars indicate standard error (SEM). Statistical analyses were performed using GraphPad Prism 9 software (GraphPad, La Jolla, CA, USA). Although *p* < 0.05 was defined as significant *a priori*, *p* ≤ 0.2 are presented in the graphs due to the conservative comparisons used.

GSIS values were compared between the highest vs. lowest quartiles (urine samples with lowest vs. highest % insulin secretion compared to control) for each compound class. GSIS values were compared between the highest vs. lowest quartiles by *t*-tests, with the Holm-Sidak method to control for multiple comparisons (1 family per GSIS response measure, 4 *t*-tests: 1 per compound class), with the overall family a = 0.05. Each compound class was analyzed individually, without assuming a consistent SD. Concentrations of each analyte class were compared between the highest vs. lowest quartiles by *t*-tests, with the Holm-Sidak method to control for multiple comparisons (1 family per compound class; 6 *t*-tests: 1 per GSIS measure). Within each compound class, GSIS values were compared between cells exposed to the highest vs. lowest quartiles of analyte concentration by *t*-tests, with the Holm-Sidak method to control for multiple comparisons (1 family per compound class, 4 *t*-tests: 1 per GSIS response metric). Each GSIS measure was analyzed individually, without assuming a consistent SD. One animal each from the vehicle and vehicle/Abx groups was euthanized due to poor response to gavage, and thus the final sample sizes are *n* = 4 for those treatments and *n* = 5 for all others.

## 3. Results

### 3.1. Flavanol Type and Abx Treatment Modify Bioavailable Flavanol and Metabolite Profiles

In our previous study, we collected and profiled metabolites in urine derived samples from rats supplemented with flavanols and treated with or without Abx. We profiled native flavanols and their phase-II and microbial metabolites to approximate total flavanol bioavailability, as most native compounds and their phase-II and microbial metabolites are excreted in the urine within 24-h of intake. We previously reported these profiles and concentration data, including individual compound concentrations [22]. This data, organized by compound class, are presented graphically in Supplementary Figure S3 (reproduced with permission from Taylor & Francis Group, Milton, United Kingdom). Pie charts showing the distribution of compounds are shown in Supplementary Figures S4–S6. Briefly, C/EC and GSE treatments exhibited similar native flavanols levels, which were significantly elevated compared to the vehicle control (Supplementary Figure S3A). However, Abx treatment did not affect native flavanol levels between C/EC and GSE. Similarly, the profiles of C/EC and GSE treatments did not differ in metabolites, and both were significantly enriched in flavanol-derived metabolites compared to vehicle control (Supplementary Figure S3B). As expected, Abx treatment completely reversed formation metabolites and levels in rats administered C/EC and GSE did not differ from the vehicle controls. While we note here that flavanol and Abx treatment affected some metabolite classes in the urine derived samples, a full discussion of urine profiles is beyond the scope of the present report and was discussed previously [22].

In the present study, we treated INS-1 832/13 β-cells with these flavanol metabolite-rich urinary extract for 24-h and measured classic GSIS parameters: unstimulated insulin secretion, stimulated insulin secretion, insulin secretion fold induction, and insulin content. We analyzed the GSIS data in three distinct ways: a treatment-level comparison, an individual-level dose response, and a responder vs. non-responder analysis of metabolite composition and GSIS. We hypothesized that among animals not treated with Abx, the metabolites from the high-molecular weight flavanol GSE would have the most beneficial bioactivity, followed by those from the low-molecular weight monomer flavanols C/EC, and then the vehicle control diet. We expected a loss of bioactivity across all Abx treated animals due to the diminished microbiome capacity to generate metabolites. Finally, like other microbiome studies, we anticipated some level of individualized responses due to unique microbiome variation in the outbred rats. 

### 3.2. Treatment-Level Comparisons: Urine Metabolites Increase β-Cell Insulin Secretion during Low Glucose Condition

**Figure 1:**
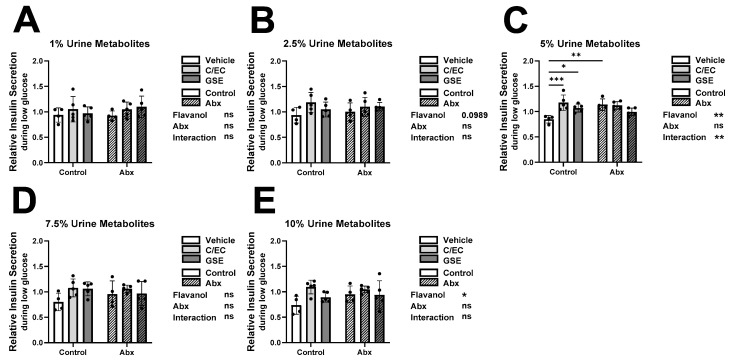
Treatment-level Comparisons show Urine Flavanol Metabolites Increase β-cell Insulin Secretion during Low Glucose Condition. Low glucose (2.8 mM) insulin secretion results of INS-1 832/13 β-cells following 24-h culture with metabolites from rats fed the vehicle (white bars), catechin/epicatechin (C/EC) (light gray bars), or grape seed extract (GSE) (dark gray bars) and treated with (striped bars) or without antibiotics (Abx) (solid bars). Urine flavanol metabolites were diluted in media at 1% (**A**), 2.5% (**B**), 5% (**C**), 7.5% (**D**), and 10% (**E**) final concentrations. Values are reported relative to the control β-cells cultured with water (Supplemental Figure S2). Data represent the average of 3 β-cell culture triplicates for each animal (n = 4–5 animals). * Represent 2-way ANOVA with Šidák’s post hoc test results of flavanol effects, Abx effects, interaction effects, and significance compared to the vehicle control. * *p* < 0.05, ** *p* < 0.01, *** *p* < 0.001 or not significant (ns).

In the first step of the GSIS assay, we measured the insulin secretion of β-cells treated with urine-derived metabolites during unstimulated conditions (Figure 1), where healthy β-cells should secrete low levels of insulin. We hypothesized that flavanol metabolites would beneficially reduce insulin secretion while loss of these metabolites by Abx treatment would inappropriately increase insulin secretion or have no effect. In this treatment-level analysis, β-cells cultured with 1%, 2.5% and 7.5% concentrations of urine metabolites showed no significant difference across treatment groups (Figure 1A,B,D) while the flavanol factor showed a significant effect in the 10% culture (Figure 1E). As hypothesized, β-cells cultured with the 5% concentration of metabolites from the vehicle and Abx treated animals detrimentally increased insulin secretion compared to the vehicle only control (Figure 1C). In contrast, the metabolites from the C/EC and GSE supplemented animals surprisingly also showed a detrimental increase in the absence of Abx treatment (Figure 1C). Therefore, we observed detrimental Abx and flavanol effects when animal treatments were grouped for analysis.

### 3.3. Metabolites Do Not Affect Insulin Secretion or Content during High Glucose Conditions

**Figure 2:**
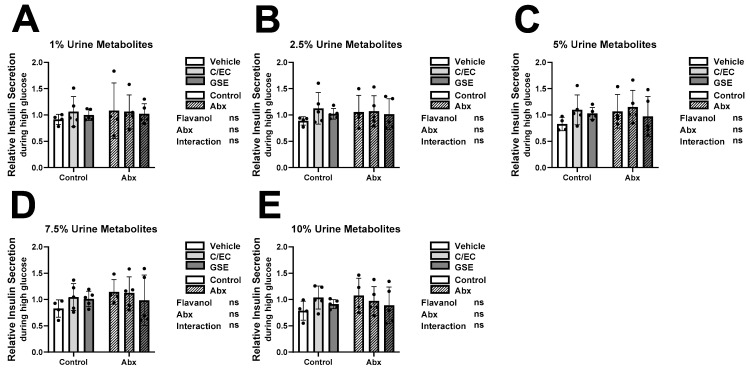
Metabolites Do Not Affect Insulin Secretion during High Glucose Condition. High glucose (12 mM) insulin secretion results of INS-1 832/13 β-cells following 24-h culture with metabolites from rats fed the vehicle (white bars), catechin/epicatechin (C/EC) (light gray bars), or grape seed extract (GSE) (dark gray bars) and treated with (striped bars) or without antibiotics (Abx) (solid bars). Metabolites were diluted in media at 1% (**A**), 2.5% (**B**), 5% (**C**), 7.5% (**D**), and 10% (**E**) final concentrations. Values are reported relative to the control β-cells cultured with water (Supplemental Figure S2). Data represent the average of 3 β-cell culture triplicates for each animal (*n* = 4–5 animals). Represent 2-way ANOVA with Šidák’s *post hoc* test results of flavanol effects, Abx effects, interaction effects, and significance compared to the vehicle control. Not significant (ns).

**Figure 3:**
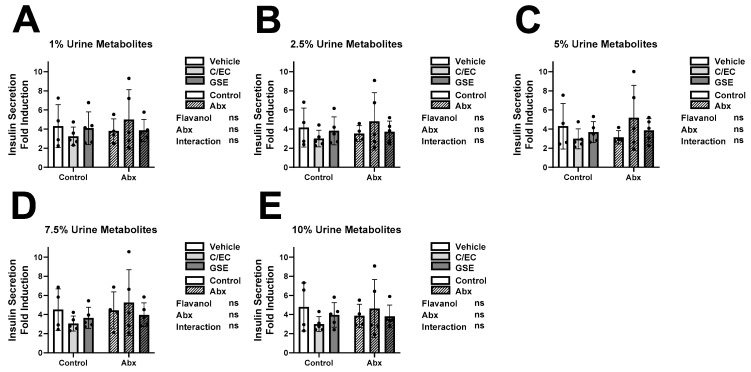
Metabolites Do Not Affect Insulin Secretion Fold Induction. Insulin secretion fold induction difference between high (Figure 2) and low (Figure 1) glucose stimulation results of INS-1 832/13 β-cells following 24-h culture with metabolites from rats fed the vehicle (white bars), catechin/epicatechin (C/EC) (light gray bars), or grape seed extract (GSE) (dark gray bars) and treated with (striped bars) or without antibiotics (Abx) (solid bars). Metabolites were diluted in media at 1% (**A**), 2.5% (**B**), 5% (**C**), 7.5% (**D**), and 10% (**E**) final concentrations. Values are reported relative to the control β-cells cultured with water (Supplemental Figure S2). Data represent the average of 3 β-cell culture triplicates for each animal (*n* = 4–5 animals). Represent 2-way ANOVA with Šidák’s *post hoc* test results of flavanol effects, Abx effects, interaction effects, and significance compared to the vehicle control. Not significant (ns).

**Figure 4:**
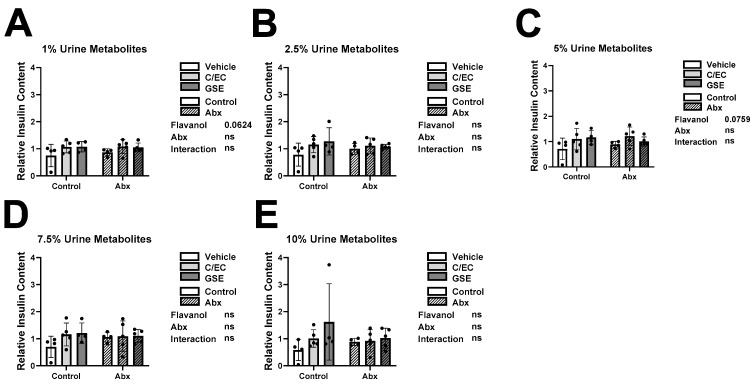
Metabolites Do Not Affect Insulin Content. Insulin content values of INS-1 832/13 β-cells following 24-h culture with metabolites from rats fed the vehicle (white bars), catechin/epicatechin (C/EC) (light gray bars), or grape seed extract (GSE) (dark gray bars) and treated with (striped bars) or without antibiotics (Abx) (solid bars). Reconstituted urine metabolites containing metabolites were diluted in media at 1% (**A**), 2.5% (**B**), 5% (**C**), 7.5% (**D**), and 10% (**E**) final concentrations. Values are reported relative to the control β-cells cultured with water (Supplemental Figure S2). Data represent the average of 3 β-cell culture triplicates for each animal (*n* = 4–5 animals). Represent 2-way ANOVA with Šidák’s *post hoc* test results of flavanol effects, Abx effects, interaction effects, and significance compared to the vehicle control. Not significant (ns).

In healthy β-cells, elevated insulin secretion and production is stimulated by increased blood glucose [52,53]. To investigate glucose response capacity of β-cells cultured with metabolites, we measured their insulin secretion rates during high glucose, or stimulatory conditions (Figure 2) and calculated the relative induction (fold increase) from low to high glucose conditions (Figure 3). We also measured their insulin content as an indirect report of their insulin production (Figure 4). Due to high variability between individuals, we observed no significant main effects or interactions and thus *post hoc* tests to assess differences between treatment groups were not performed (Figure 2, Figure 3 and Figure 4). As expected, individual variability was much higher for high glucose insulin secretion and insulin secretion fold induction (Figure 2 and Figure 3) compared to low glucose insulin secretion (Figure 1).

### 3.4. Individual-Level Effects on GSIS Regardless of Treatment

Despite observing minimal changes in the traditional treatment-level comparisons, we saw many significant dose responses from analyses on the individual animal-level. Surprisingly, metabolites from animals in different treatment groups showed a deleteriously elevated insulin secretion during the low glucose condition (Supplementary Figure S7). Both beneficial and deleterious effects were observed on insulin secretion during high glucose (Supplementary Figure S8), fold induction (Supplementary Figure S9) and insulin content (Supplementary Figure S10). These mixed results were seemingly irrespective of animal treatment groups and at least one individual from each group presented significant individualized results. Paradoxically, even the vehicle only control group was represented (Supplementary Figure S10A). When results for all parameters are considered together, one animal did stand out as consistently supporting our hypothesis that Abx treatment would be detrimental. Metabolite cultures from animal 7 in the GSE and Abx group demonstrated reduced insulin secretion during high glucose (Supplementary Figure S8A), fold induction (Supplementary Figure S9A) and insulin content (Supplementary Figure S10B). However, conclusions about overall metabolite patterns were unclear because one third of the animals (9 of 27) showed some significant, and often contradictory, individualized response.

While some treatment-level (Figure 1, Figure 2, Figure 3 and Figure 4) and individual-level (Supplementary Figures S7–S10) results supported our original hypotheses, we observed high inter-individual variability which obscured broad treatment-level conclusions. Therefore, we postulated that the bioactivity of the urine extracts is less associated with the experimental treatment and more related to the unique individual microbiome or metabolite profile resulting from the outbred animals. Another confounding factor is that we only characterized flavanol-derived metabolites in the urine. We may have missed some key metabolites that either remain unknown or were present at concentrations too low to characterize. There are numerous unmeasured metabolites, both of flavanol origin as well as endogenous or from the bulk diet, present in the urine. This is a limitation of the present analysis. Untargeted metabolomics would address some of these differences. 

### 3.5. Responder Analyses Show Metabolite Profiles Predict GSIS Activity Independent of Treatment

**Figure 5:**
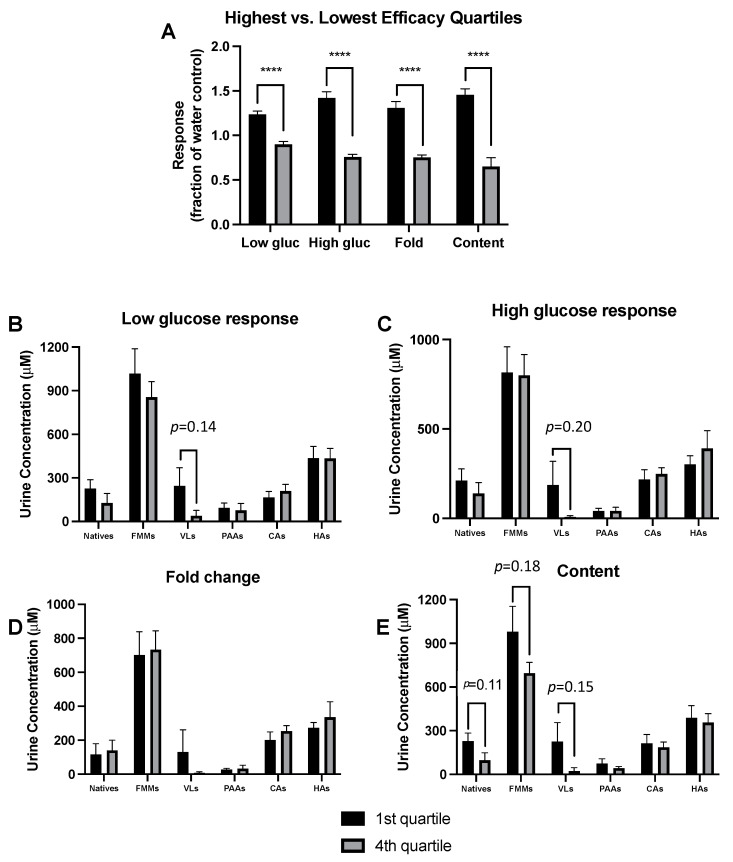
Responder vs. Non-responder Predictive Analyses of GSIS based on Metabolite Profiles. (**A**) Differentiation of urine sample extracts (5% dose only) by highest vs. lowest quartiles for GSIS parameters. GSIS was compared between the highest (black bars) vs. lowest quartiles (gray bars) by *t*-tests, with the Holm-Sidak method to control for multiple comparisons (1 family, 4 *t*-tests: 1 per GSIS response measure), with the overall family α = 0.05. Each GSIS measure was analyzed individually, without assuming a consistent SD. Comparison of urine compositions between the most effective vs. least effective samples for each GSIS measure including insulin secretion during low glucose (**B**), during high glucose (**C**), insulin secretion fold induction (**D**), and insulin content (**E**). Within each GSIS measure, levels of each compound class were compared between the highest vs. lowest quartiles by *t*-tests, with the Holm-Sidak method to control for multiple comparisons (1 family per GSIS measure, 6 *t*-tests: 1 per compound class). For all tests: **** *p* < 0.0001.

We next performed individual-level analysis of GSIS activity based on metabolite profile, independent of treatment. Due to the large number of treatments, only GSIS data from the 5% urine dose were used. We determined which samples were most vs. least effective modulators of GSIS capacity relative to the vehicle control for the GSIS parameters (irrespective of treatment) and selected the most vs. least effective quartiles (7 animals per quartile) (Figure 5A). This analysis resulted in significant separation, with low variability within quartiles, which facilitates a responder analysis not possible with the constrains of the traditional treatment-based grouping.

We next compared profiles for various compound classes of only the most vs. least effective quartiles of metabolites by compound class and GSIS parameter (Figure 5B–E). Due to the large number of quantified metabolites, individual compound data was not used for this analysis. Benzoic acids, other aromatics, and non-aromatics were also excluded due to lack of significant differences across treatments (Supplementary Figure S3G–I). This analysis resulted in some differences approaching significance. For low and high glucose responses there were no significant differences, but levels of valerolactones (a microbial metabolite unique to flavanols) approached significance between low and high responders (*p* = 0.14 and 0.20 for low and high glucose responses, respectively) (Figure 5B,C). There were no significant differences for fold induction (Figure 5D). For insulin content, the differences in composition between lowest and highest responders approached significance for total natives, microbial metabolites, and valerolactones (*p* = 0.11, 0.18 and 0.15, respectively). In all cases approaching significance, higher levels appear correlated with greater GSIS activity. Due to the exploratory *post hoc* nature of this analysis, the statistical tests were intentionally very conservative, as the overall family error rate (1 family per GSIS response, 6 *t*-tests per family, one per compound class) was set at α = 0.05 to reduce the rate of false positives; adjusted *p*-values are presented and used for evaluation of statistical significance. In a less conservative analysis, statistical significance would be achieved. This analysis suggests that native flavanols, total microbial metabolites and valerolactones are the active compounds for enhancing GSIS. These data agree with our previous publications showing that native catechins and valerolactones possess β-cell stimulatory activities [8,46].

**Figure 6:**
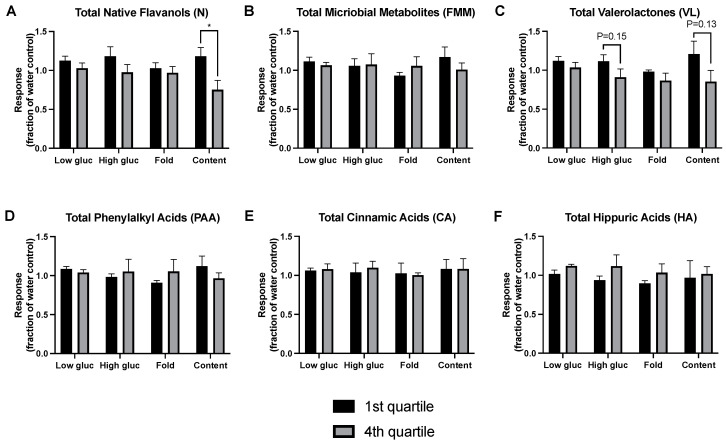
Predictive Value of Metabolite Profiles on GSIS activity. Comparison of GSIS response between urine derived samples with the highest and lowest quartiles of concentrations of various classes of compounds, including total (**A**) native flavanols, (**B**) microbial metabolites, (**C**) valerolactones, (**D**) phenylalkyl acids, (**E**) cinnamic acids and (**F**) hippuric acids. Values are presented as mean ± SEM. Within each compound class, response was compared between the highest vs. lowest quartiles by *t*-tests, with the Holm-Sidak method to control for multiple comparisons (1 family per compound class, 4 *t*-tests: 1 per response measure). * *p* < 0.05.

The findings in Figure 5 suggest that specific compound classes are associated with enhanced GSIS activity, when the highest vs. lowest efficacy urine samples were compared independent of treatment. To further evaluate these findings, the reverse analysis was performed: the highest and lowest concentration quartile of each compound class were determined, and the GSIS capacity of these urine derived samples were compared (Figure 6). This reverse analysis strengthened several results from Figure 5. For total native flavanols, the highest quartile of metabolite levels produced significantly higher insulin content levels compared to the lowest levels (Figure 6A). For valerolactones (Figure 6C), the highest vs. lowest quartile approached significance during the high glucose condition (*p* = 0.15) and insulin content (*p* = 0.13). There was no significant separation in GSIS response between quartiles for total microbial metabolites or any microbial metabolite class (Figure 6B,D–F). Again, highly conservative tests were used due to the exploratory nature of this analysis and the number of comparisons. 

## 4. Discussion

Type 2 Diabetes has historically been defined as a disease characterized by muscle, adipose and liver insulin insensitivity with lost functional β-cell mass, including dysregulated GSIS being a later occurrence resulting from long-term insulin insensitivity. However, recent findings strongly demonstrate that defective insulin secretion may be an instigating factor in Type 2 Diabetes diseases progression. While the effect of flavanols on muscle, adipose and liver have historically been more studied [16], in recent years studies have begun to look at the effect of flavanols on β-cell proliferation, insulin secretion and survival [33,40,41]. While early studies of flavanols and β-cell proliferation, survival, and insulin secretion focused on in vivo feeding studies and treatment of β-cells with native flavanols in vitro [33,40,41], we have shown that epicatechin [46] and three of its metabolites (hippuric acid, homovanillic acid, and 5-phenylvaleric acid) individually are sufficient to enhance GSIS in INS-1 832/13 β-cells [8]. While epicatechin enhances GSIS by increasing mitochondrial respiration and mitochondrial electron transport chain component expression [46], our published data demonstrates that hippuric acid, homovanillic acid, and 5-phenylvaleric acid increase insulin secretion and insulin content without modulating mitochondrial electron transport chain component levels or modulating mitochondrial respiration [8]. These data suggest that gut microbiome derived metabolites may be sufficient to enhance GSIS, indicating that microbial metabolites may mediate some of the observed metabolic effects in flavanol consumption studies. Furthermore, with our previous published data, our findings suggest that the method of action may be significantly different from that induced by the native flavin. However, this represents isolated β-cells only, and the whole-body effects of these metabolites on other complementary tissues cannot be determined in this model. The purpose of the present study was to move beyond isolated native flavanols and microbial metabolites and study the physiologically relevant complex mixtures of bioavailable native flavanols and their metabolites that were produced and absorbed in rats gavaged with either simple monomeric flavanols or GSE containing larger flavanols on insulin secretion.

Looking at the compiled data, our findings show that the urine derived metabolites from the C/EC- or GSE-gavaged rats increased unstimulated insulin secretion while samples from Abx treated animals had no change. The potential implications of increased unstimulated insulin secretion in low glucose conditions by flavanols remain unclear and warrant further consideration. As mentioned above, the targeted nature of the present analysis may have omitted key metabolites that may explain this effect. We observed no changes in stimulated insulin secretion, fold insulin secretion, or insulin content when averaged across groups. Interestingly, our data did demonstrate variability in the INS-1 832/13 β-cell response based on the individual rat source of the urine derived metabolites, independent of treatment. These data demonstrate an animal specific effect, based on the metabolites found in the urine, specifically looking at total native flavanols on insulin content. Similarly, the concentration of valerolactons appeared to have the strongest effect, comparing quartile 1 and quartile 4, on unstimulated, stimulated, and fold insulin secretion, as well as insulin content. These differences, which appear to reflect the individual microbiome composition, are supported by our previous findings demonstrating significant sex based and microbiome-based differences in terms of response to EC [54]. Overall, these findings suggest that native flavanols and valerolactones, but not the smaller metabolites (such as phenylpropionic or acetic acids, cinnamic acids, hippuric acids, benzoic acids, etc.), appear to exert some effect on increasing insulin secretion capacity by β-cells.

Our findings may also be impacted by the complex nature of the urine derived metabolites. While we have previously shown beneficial effects of single metabolites on insulin secretion and other metabolic processes of β-cells [8,46], the use of complex, physiologically relevant in vivo-derived metabolites allows for combinatorial effects that are dose dependent. Within the milieu of metabolites there may be compounds that activate and compounds that inhibit insulin secretion and content. Understanding the individual effects, as well as the combinatorial effects, is extremely important. Alternatively, the use of metabolites found in the urine may select against metabolites that are bound to and removed from circulation by β-cells, or other tissues. The ability to do a similar study looking at postabsorptive serum metabolites would significantly strengthen the findings from the present study [55].

The high inter-individual variability between animals within treatment groups posed the greatest challenge to interpreting the present data, precluding conclusions at the treatment level. Some steps were taken to reduce variability. We fed all rats a uniform total dose and reconstituted urine extracts to a uniform volume to normalize concentration differences across animals due to factors such as body size, urine volume, etc. This eminimized the effect of body mass or urine volume. Urine extract identities were blinded to individuals performing the in vitro cell culture assays, and β-cell culture and assay protocols were highly standardized a priori and performance was harmonized through careful staff training. At the data analysis level, all calculations were relative to within-experiment controls to enable comparisons between all experiments regardless of differences that would arise from small differences in the passage number of β-cells used in each experiment. Nevertheless, significant inter-individual variability was observed in chemical composition and in vitro activity between urine samples from animals within treatment groups. One major source of inter-individual variation was the choice of rodent model. While using outbred rats is a standard approach in bioavailability research, inbred animals may be required to reduce variability to isolate and study treatment-level effects. Alternatively, urine samples could be pooled for replicate animals with a treatment to reduce variability. Another sources of variation, particularly in metabolite production, may have been microbiome differences. Humanization of rats from a single common fecal sample of human origin could further reduce individual variability. These approaches will be explored in future studies. In response to this high interindividual variability, we explored compound-activity associations in a relatively novel approach by analyzing the responders vs. non-responders, independent of treatment. This analysis revealed that elevated levels of bioavailable native flavanols and valerolactones were associated with improved beta cell function. Due to the exploratory nature of the responder analysis and large number of comparisons, conservative post hoc tests were used, and these results (adjusted *p*-values) thus approached but did not reach significance.

There are several limitations of the present work. First, we used urine as the source of physiologically relevant sources of metabolite mixtures based on the logic that all flavanol-derived compounds in urine must be present in the bloodstream. While we took precautions to reduce the content of salts and urea and included a vehicle group to account for the urine background, the fact remains that β-cell are never exposed to actual urine in vivo, and thus there are potential effects in this model that do not occur in vivo. Additionally, flavanol exposure may have induced secretion of other factors that were present in the urine that contribute to beta cell function, and this is a limitation of the present design. Use of serum for similar studies has been performed [55,56] but presents challenges of low sample volumes. Such strategies may be required in the future. Furthermore, urine is an accumulated sample and does not represent the natural pharmacokinetic concentration and profile changes that occur in circulating bioavailable compounds over time post-dose. Second, the observed inter-individual variability in urine profiles and activities was much larger than we expected, as discussed above. Third, the flavanols treatments may not have differed enough to produce the desired separation in treatment effects. We know that some of these metabolites can come from the background diet, even highly purified diets, and thus some variability and non-treatment background effect in metabolite composition was unavoidable regardless of the treatments selected [22,57]. While the C/EC treatment had only monomeric flavanols, the GSE treatment contained monomeric as well as larger flavanols. The overlap in composition (monomeric flavanols in both groups) may have muddled the result. A cleaner design would have been to eliminate one of the flavanol treatments or compare C/EC vs purified oligomers/polymers. However, standards of purified oligomeric/polymeric flavanols are not economically available in the large quantities needed for the gavage studies. Finally, these studies were all done under optimal culture conditions. β-cell insulin secretion is impaired by elevated blood glucose or culture with fatty acids [45]. While the current study looked at the ability of these metabolites to modulate GSIS under optimal conditions, use of glucoplipotoxic conditions would have permitted us to explore the ability of these compounds to modulate GSIS under suboptimal conditions. Future studies will need to determine if these metabolites are sufficient to ameliorate impaired GSIS under suboptimal conditions. 

In conclusion, this study demonstrates that the profile of bioavailable flavanol-derived metabolites is highly variable, as is the subsequent effect of these mixtures on β-cell function. Bioavailable mixtures obtained following flavanol administration appeared to enhance insulin secretion under low-glucose (unstimulated) conditions, which warrants further study. The effects on beta cells under high-glucose (stimulated) conditions was more variable between individuals, precluding conclusions by treatment groups. However, when the most vs. least active samples and the highest vs. lowest metabolite concentrations were studied independent of treatment group, associations between higher levels of flavanols and valerolactones and increased β-cell function approached significance. While not conclusive, the present study provides hypothesis-generating data and new experimental protocols that can be used to further investigate the role of bioavailable flavanol-derived metabolites in protecting β-cell health and function in vivo. 

## Data Availability

The data presented in this study are available in this article.

## Supplementary Materials

The following supporting information can be downloaded at: https://www.mdpi.com/article/10.3390/metabo13070801/s1, Figure S1: Dietary Flavanol Structures, Figure S2: Insulin Secretion Fold of Water Controls, Figure S3: Concentrations of total levels of native flavanols, flavanol microbial metabolites, and their phase-II conjugates in urine samples. Figure S4: Pie charts showing the mean distribution (fraction of total, pie charts) and total sum of native flavanols and flavanol microbial metabolites (FMMs) in urine from the six treatment groups. Figure S5: Pie charts showing the mean distribution (fraction of total) by compound class compounds and total measured compounds in urine from the six treatment groups. Figure S6: Pie charts showing the mean distribution (fraction of total) by phase-II conjugation and total measured compounds in urine from the six treatment groups. Figure S7: Individual-level Dose Responses of Metabolites from Individual Animals on β-cell Insulin Secretion under Unstimulated Conditions. Figure S8: Dose Responses of Metabolites from Individual Animals on Insulin Secretion under Stimulated Condition. Figure S9: Dose Responses of Metabolites from Individual Animals on Insulin Secretion Fold Induction. Figure S10: Dose Responses of Metabolites from Individual Animals on Insulin Content. Table S1: Composition of Vitaflavan grape seed extract.
