# Supplementary material for: Bioavailable Microbial Metabolites of Flavanols Demonstrate Highly Individualized Bioactivity on In Vitro β-Cell Functions Critical for Metabolic Health
Source: Metabolites. 2023 Jun 28;13(7):801. doi: 10.3390/metabo13070801 (PMC10385630; doi:10.3390/metabo13070801)
Supplement: Supplementary file 1 [file metabolites-13-00801-s001.zip › metabolites-2467277-supplementary.pdf]

Supplementary

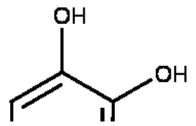

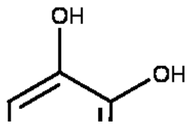

(+)-catechin (−)-epicatechin

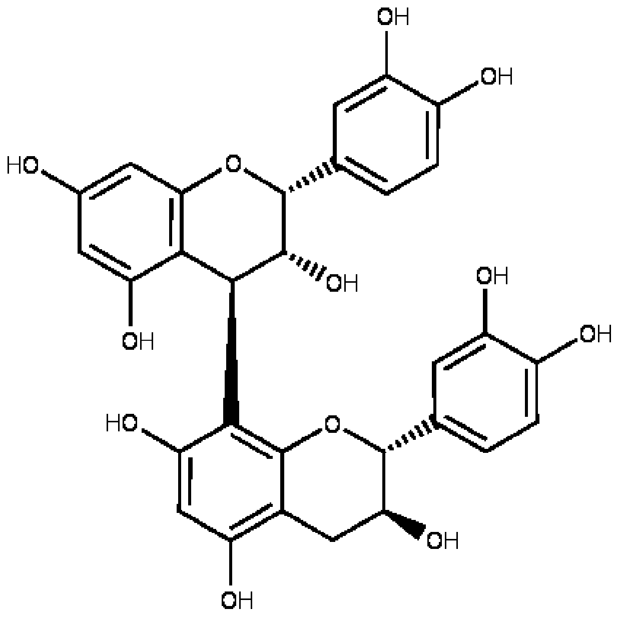

procyanidin B2

Supplemental Figure S1 Dietary Flavanol Structures

Structures of flavanols commonly found in the human diet from sources such as cocoa, tea, fruits, etc.

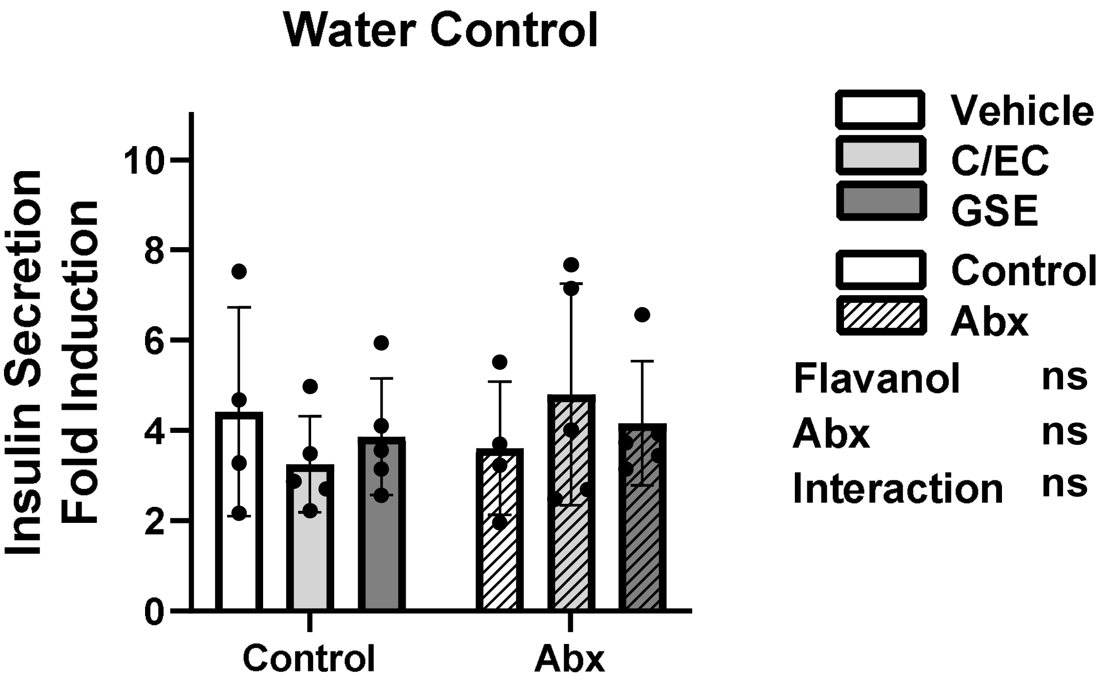

Supplemental Figure S2 Insulin Secretion Fold of Water Controls.

Insulin secretion fold induction difference between high and low glucose stimulation results of INS-1 832-13 β-cells following 24-hour culture with 10% water controls for comparison with results in Figure 3 for urine metabolites from rats fed the vehicle (white bars), catechin/epicatechin (C/EC) (light gray bars), or grape seed extract (GSE) (dark gray bars) and treated with (striped bars) or without antibiotics (Abx) (solid bars). Data represent the average of 3 β-cell culture triplicates for each animal (*n*=4-5 animals. *Represent 1-way ANOVA with Dunnett's post hoc test results show flavanol effects, Abx effects, interaction effects, and significance compared to the vehicle control. Not significant (ns).

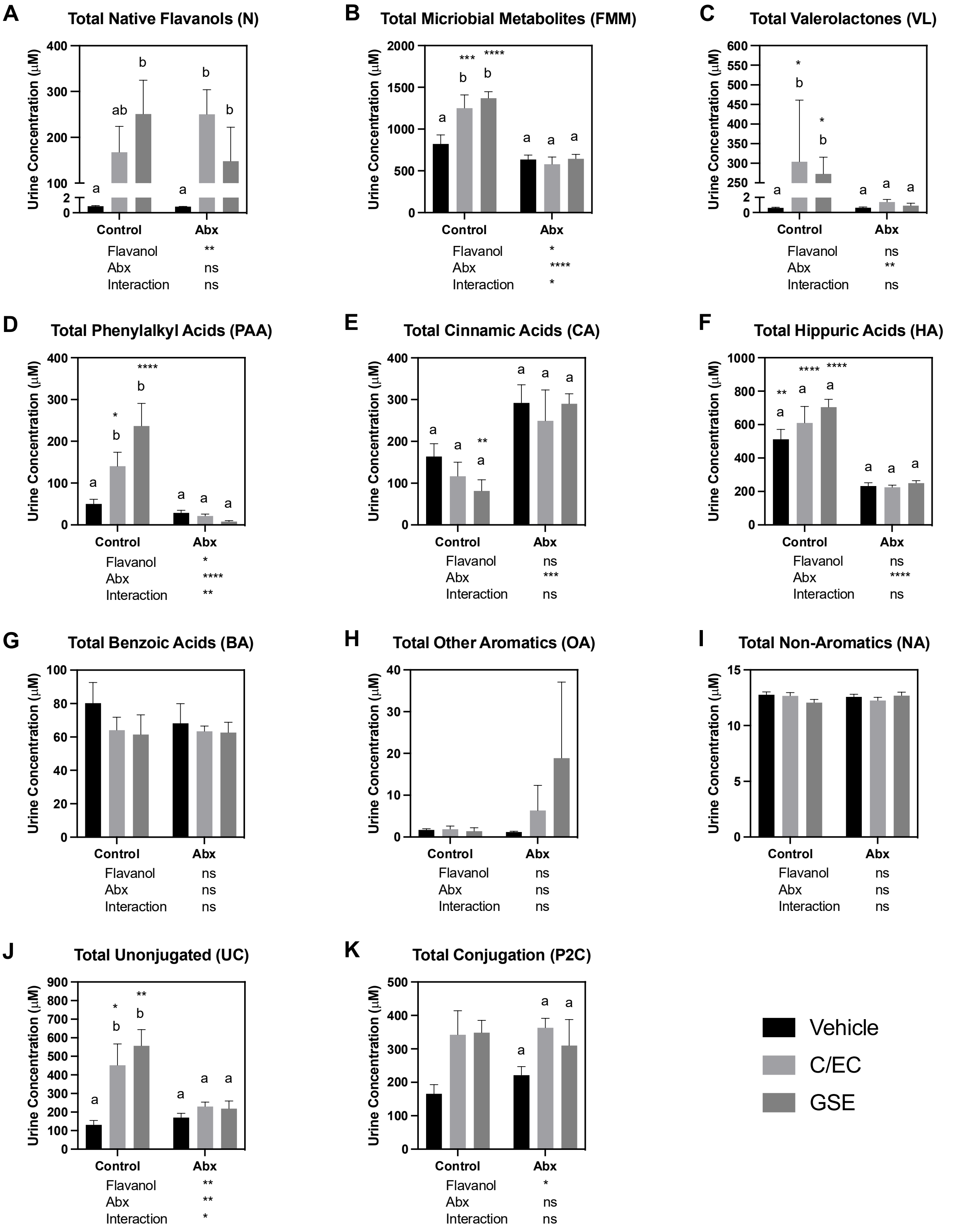

Supplementary Figure S3. Concentrations of total levels of native flavanols, flavanol microbial metabolites, and their phase-II conjugates in urine samples. Values are presented as mean ± SEM. For each measure, 2-way ANOVA was performed to determine the statistical significance

of main effects (flavanol and Abx treatment) and interactions. Values below each graph indicate the results of 2-way ANOVA. If a significant main effect or interaction was detected, Holm-Sidak post hoc tests to account for multiple comparisons were performed to determine differences among the 3 flavanol treatments within each antibiotic treatment group (Control and Abx); bars not sharing a common superscript letter within each group are statistically different. Holm-Sidak post hoc tests were also performed to determine differences between antibiotic treatments (Control and Abx) for each flavanol; asterisks indicate a significant difference for that flavanol between Control and Abx (*P<0.05, **P<0.01, ***P<0.001, ****P<0.0001). The overall family-wise error rate was set as 0.05, with one family per group. This figure originally appeared as Figure 2 in Griffin LE, SE Kohrt, A Rathore, CD Kay, MM Grabowska, AP Neilson, Microbial metabolites of flavanols in urine are associated with enhanced anti-proliferative activity in bladder cancer cells *in vitro*, *Nutr Cancer* 2021; 74(1):194-210. https://doi.org/10.1080/01635581.2020.1869277. PMID: 33522303 (https://pubmed.ncbi.nlm.nih.gov/33522303/). Preprint (bioRχiv) DOI: https://doi.org/10.1101/2020.09.22.308056. See www.tandfonline.com. Reproduced with permission of Taylor & Francis Ltd. Permission granted 12 Oct 2022. Abbreviations: V: vehicle, Abx: antibiotics, C/EC: catechin/epicatechin, GSE: great seed extract

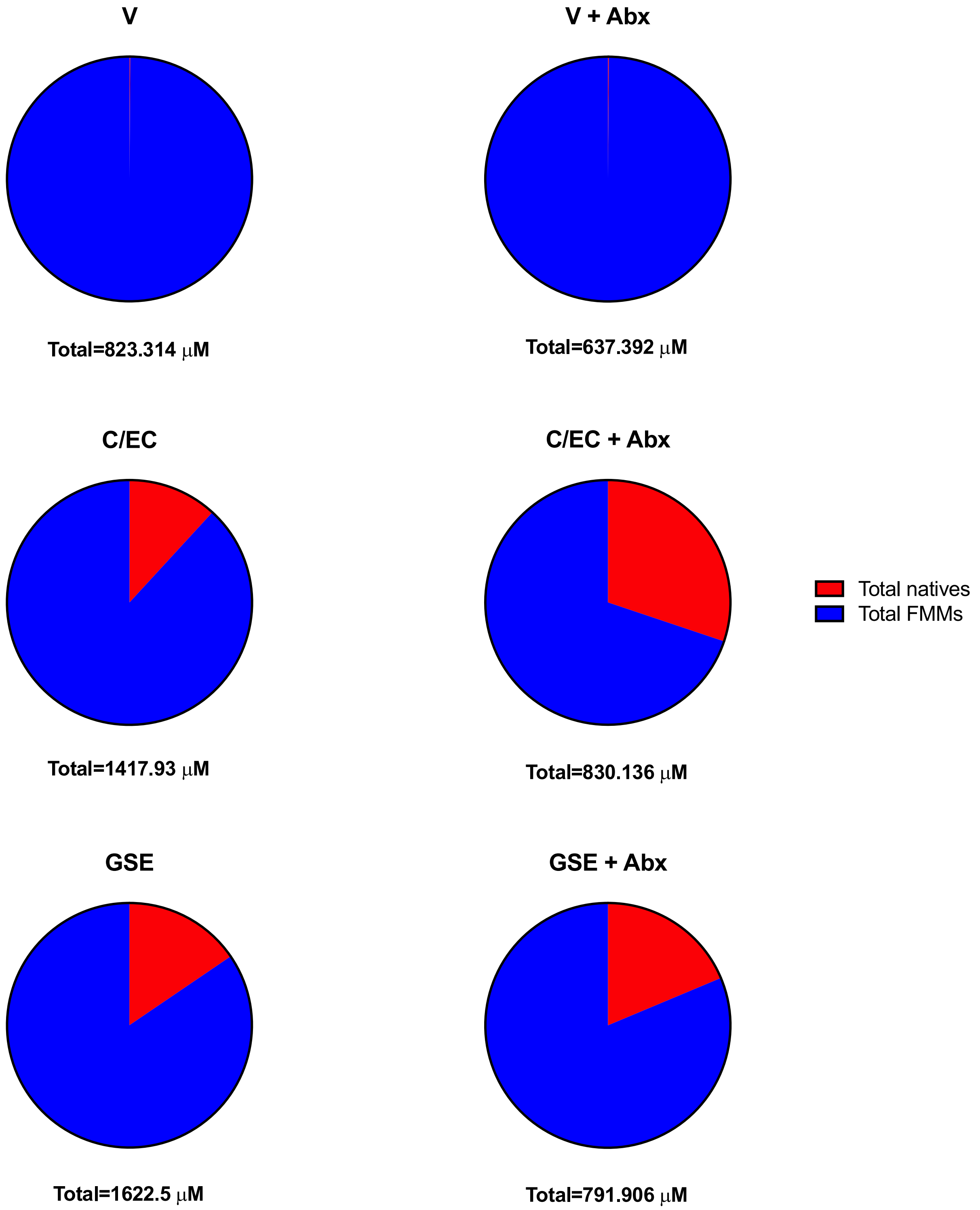

Supplementary Figure S4. Pie charts showing the mean distribution (fraction of total, pie charts) and total sum of native flavanols and flavanol microbial metabolites (FMMs) in urine from the six treatment groups. Abbreviations: V: vehicle, Abx: antibiotics, C/EC: catechin/epicatechin, GSE: great seed extract.

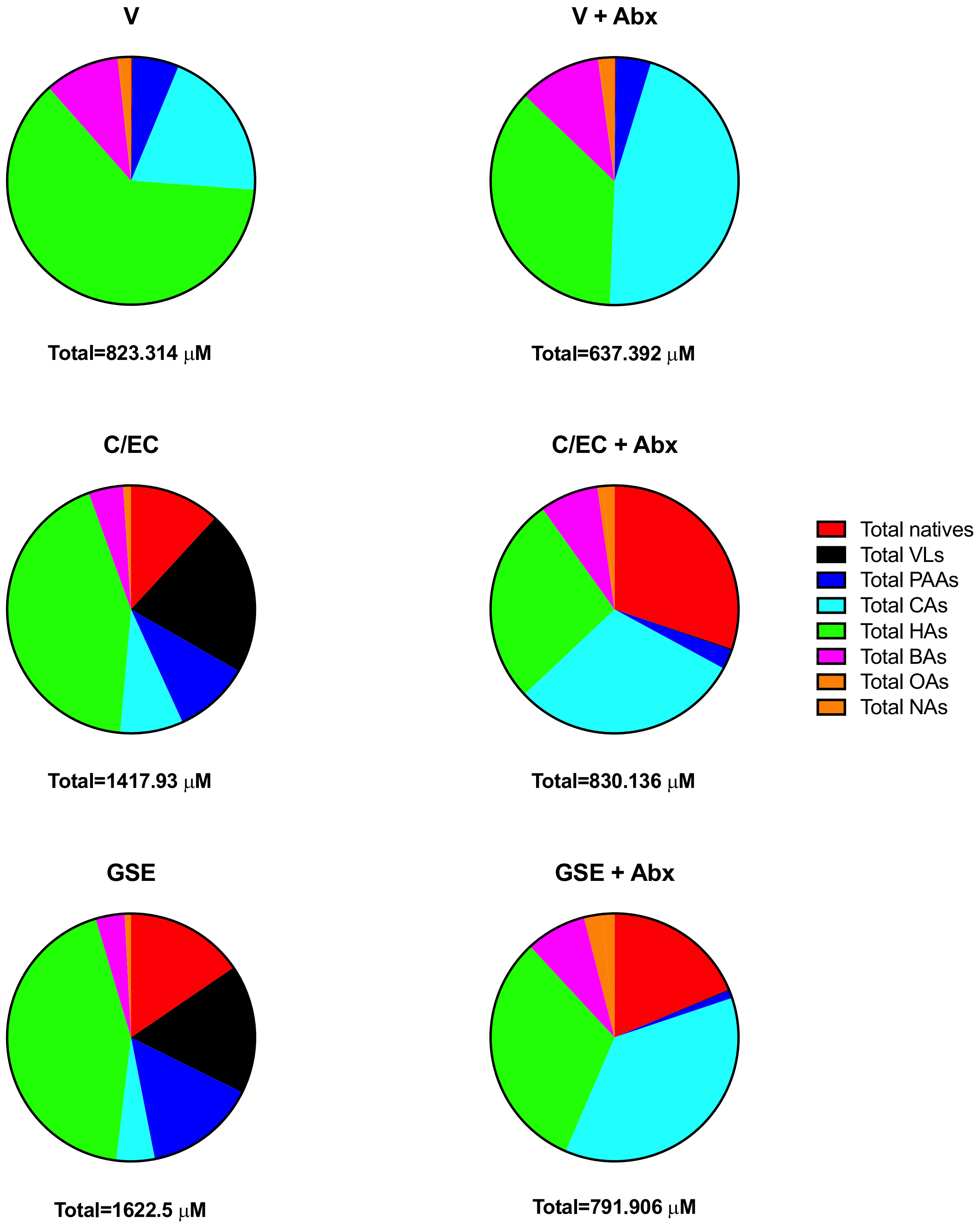

Supplementary Figure S5. Pie charts showing the mean distribution (fraction of total) by compound class compounds and total measured compounds in urine from the six treatment groups. Abbreviations: V: vehicle, Abx: antibiotics, C/EC: catechin/epicatechin, GSE: great seed extract

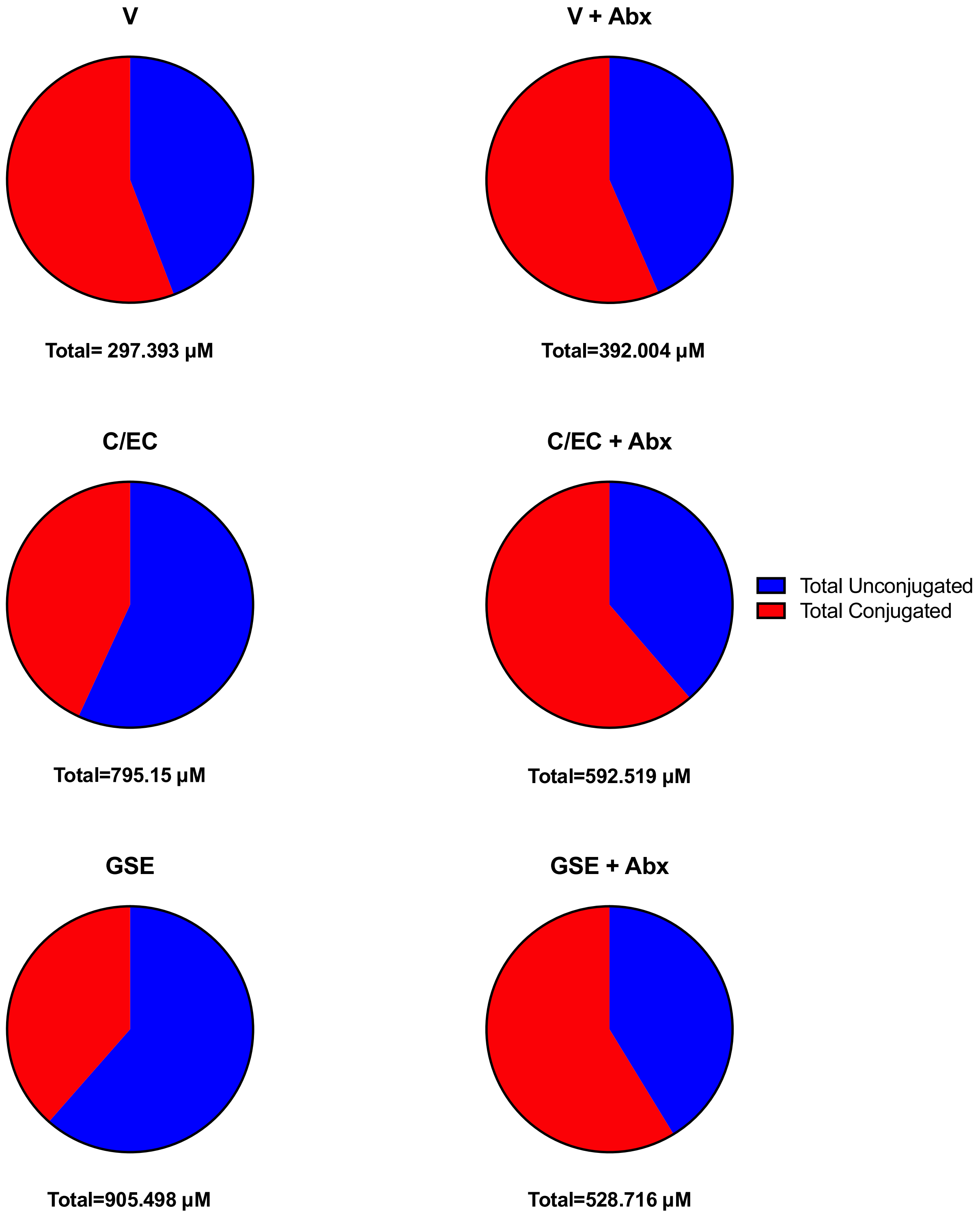

Supplementary Figure S6. Pie charts showing the mean distribution (fraction of total) by phase-II conjugation and total measured compounds in urine from the six treatment groups. Abbreviations: V: vehicle, Abx: antibiotics, C/EC: catechin/epicatechin, GSE: great seed extract

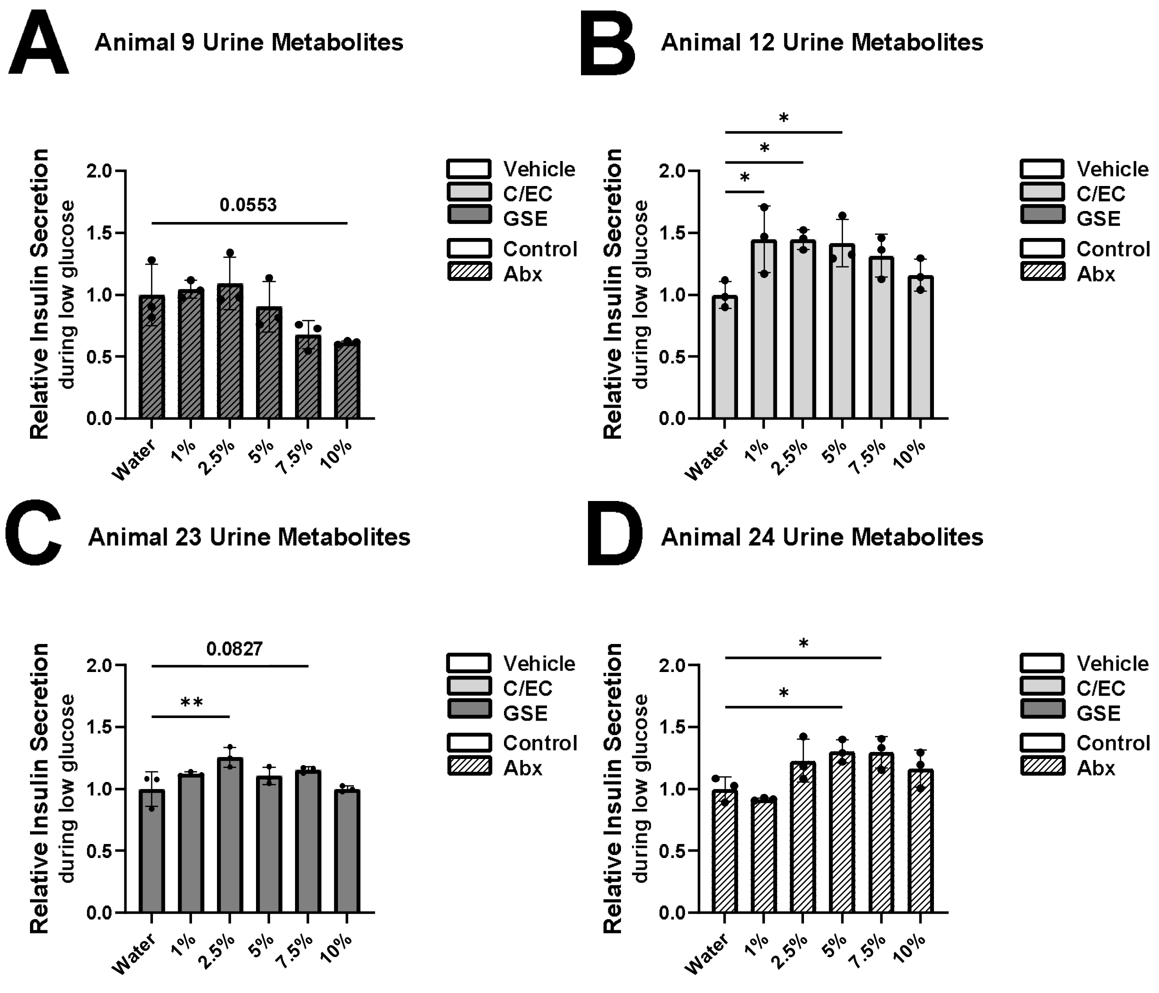

Supplementary Figure S7: Individual-level Dose Responses of Metabolites from Individual Animals on β-cell Insulin Secretion under Unstimulated Conditions.

Low glucose insulin secretion results of INS-1 832/13 β-cells cultured with metabolites of 4 individual rats from Figure 1 showing significant dose response effects. Dose responsive rats were fed GSE with Abx (A), C/EC (B), GSE (C), and Vehicle with Abx (D). Metabolites were diluted in media at 1%-10% final concentrations. Values are reported relative to the control β-cells cultured with water. Data represent the average of 3 β-cell culture triplicates for each animal. *Represent 1-way ANOVA with Dunnett's *post hoc* test results of significant dose effects compared to the water control. *$p<0.05$, **$p<0.01$, or not significant (ns). Abbreviations: V: vehicle, Abx: antibiotics, C/EC: catechin/epicatechin, GSE: great seed extract

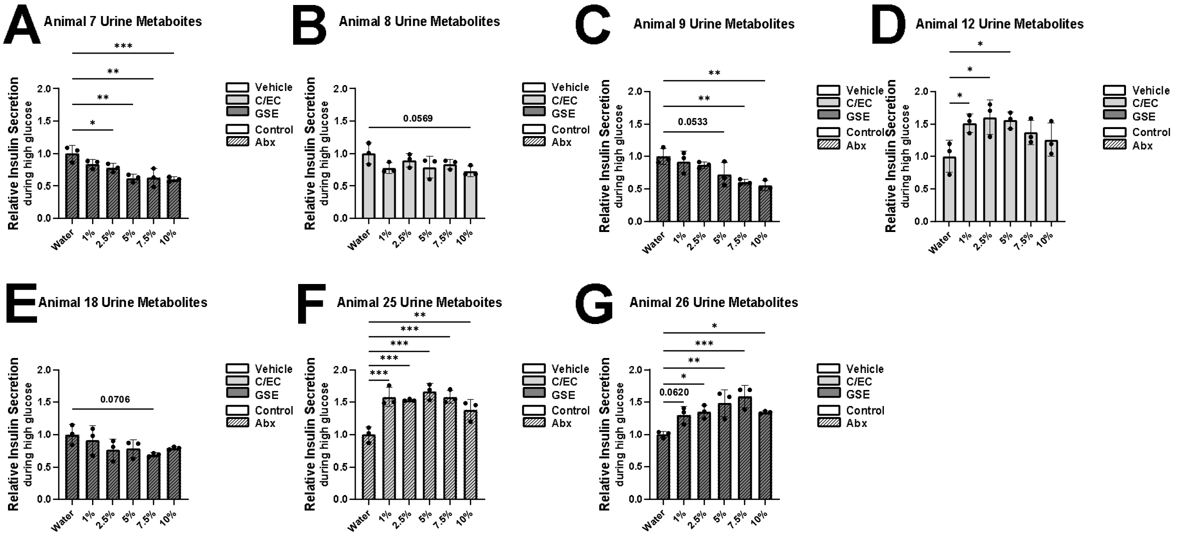

Supplementary Figure S8: Dose Responses of Metabolites from Individual Animals on Insulin Secretion under Stimulated Condition

High glucose insulin secretion results of INS-1 832/13 β-cells cultured with metabolites of 7 individual rats from Figure 2 showing significant dose response effects. Dose responsive rats were fed GSE with Abx (A,C,E,G), C/EC (B,D), and C/EC with Abx (F). Metabolites were diluted in media at 1%-10% final concentrations. Values are reported relative to the control β-cells cultured with water. Data represent the average of 3 β-cell culture triplicates for each animal. *Represent 1-way ANOVA with Dunnett's *post hoc* test results of significant dose effects compared to the water control. *p<0.05, **p<0.01, ***p<0.001 or not significant (ns).
Abbreviations: V: vehicle, Abx: antibiotics, C/EC: catechin/epicatechin, GSE: great seed extract

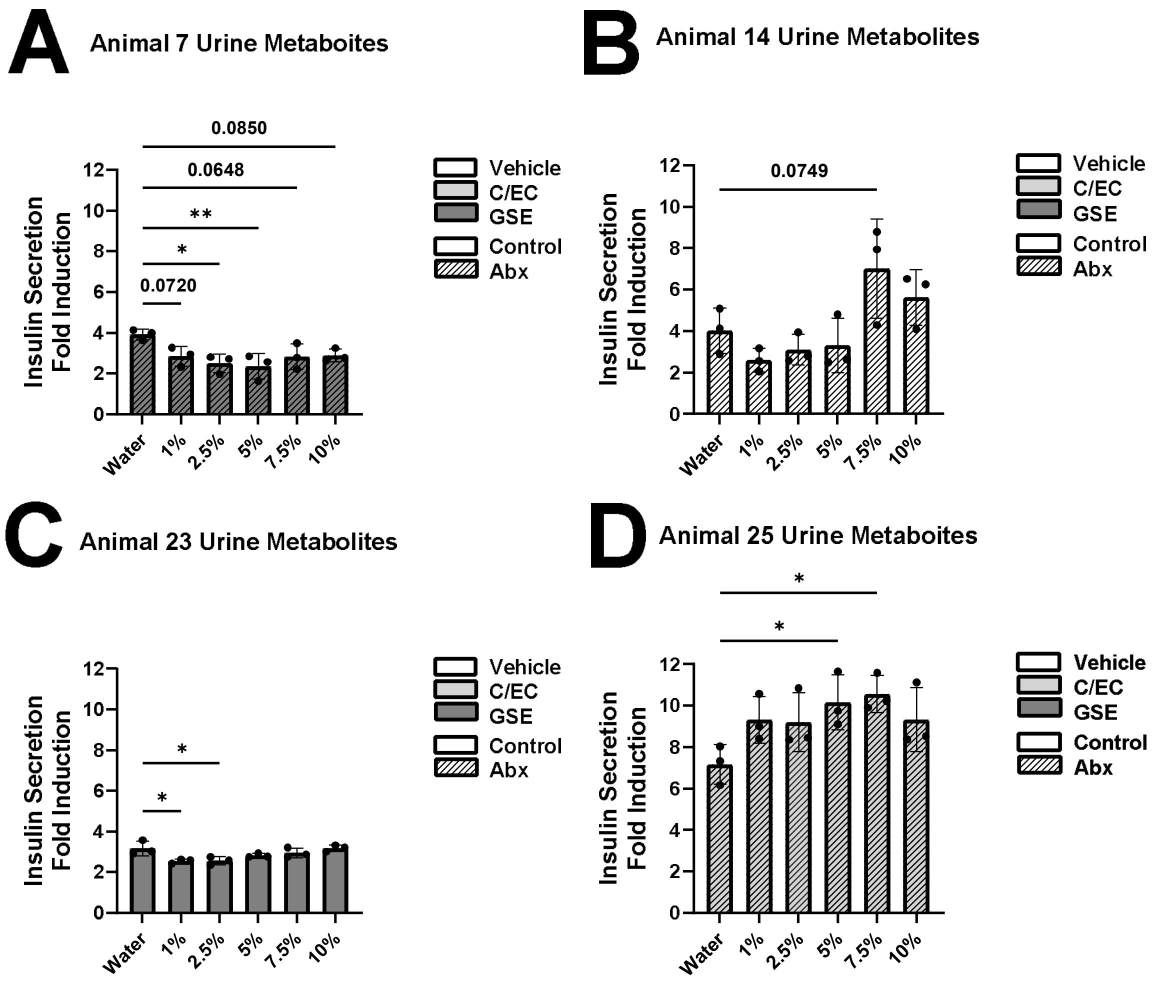

Supplementary Figure S9: Dose Responses of Metabolites from Individual Animals on Insulin Secretion Fold Induction

Insulin secretion fold induction results of INS-1 832/13 β-cells cultured with metabolites of 4 individual rats from Figure 3 showing significant dose response effects. Dose responsive rats were fed GSE with Abx (A), Vehicle with Abx (B), GSE (C), and C/EC with Abx (D). Metabolites were diluted in media at 1%-10% final concentrations. Values are reported relative to the control β-cells cultured with water. Data represent the average of 3 β-cell culture triplicates for each animal. *Represent 1-way ANOVA with Dunnett's *post hoc* test results of significant dose effects compared to the water control. *$p<0.05$, **$p<0.01$, or not significant (ns). Abbreviations: V: vehicle, Abx: antibiotics, C/EC: catechin/epicatechin, GSE: great seed extract

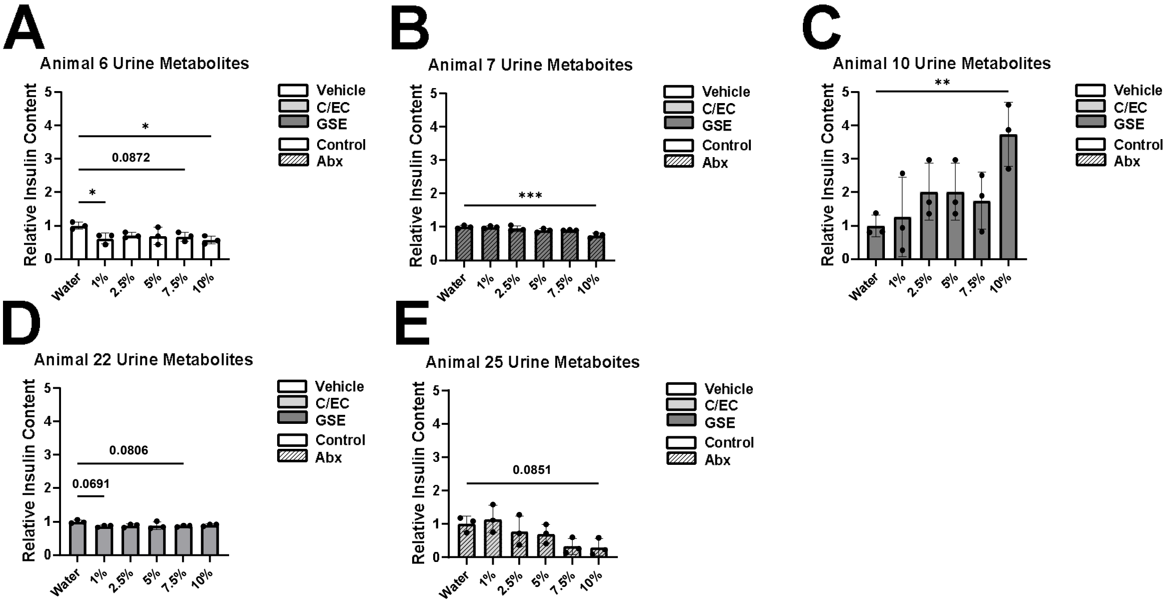

Supplementary Figure S10: Dose Responses of Metabolites from Individual Animals on Insulin Content

Insulin content results of INS-1 832/13 β-cells cultured with metabolites of 5 individual rats from Figure 4 showing significant dose response effects. Dose responsive rats were fed the Vehicle (A), GSE with Abx (B), GSE (C), (D), and C/EC with Abx (E). Values are reported relative to the control β-cells cultured with water. Data represent the average of 3 β-cell culture triplicates for each animal. *Represent 1-way ANOVA with Dunnett's *post hoc* test results of significant dose effects compared to the water control. *p<0.05, **p<0.01, ***p<0.001 or not significant (ns). Abbreviations: V: vehicle, Abx: antibiotics, C/EC: catechin/epicatechin, GSE: great seed extract

Supplementary Table S1. Composition of Vitaflavan®[1] grape seed extract

Total Polyphenol Content	>96%
Flavanol monomers	<25%
Flavanol Dimers + Trimers	>30%
Total Procyanidins Content	>75%
Procyanidins Content (Porter)	70

[1]Composition data provided by DRT Nutraceutics, Dax, France
